# The Inhibitory Effect of Clindamycin on *Lactobacillus in vitro*

**DOI:** 10.1155/S1064744901000394

**Published:** 2001

**Authors:** Alla Aroutcheva, Jose A. Simoes, Susan Shott, Sebastian Faro

**Affiliations:** 1 Department of Obstetrics and Gynecology Rush-Presbyterian-St. Luke’s Medical Center Chicago IL USA; 2 Department of Obstetrics Gynecology and Reproductive Medicine University of Texas Houston Health Science Center and The Woman’s Hospital of Texas Houston TX USA

## Abstract

*Objective:* To evaluate the *in vitro* effect of varying concentrations of clindamycin on *Lactobacillus* spp.

Methods: Concentrations of clindamycin ranging from 1.95–20 000 mg/ml were studied for their effect on the growth of six strains of *Lactobacillus* .

*Results:* Clindamycin concentrations between 1.95–31.25 mg/ml had no statistically significant effect on growth of lactobacilli (*p* > 0.05). Concentrations 125 and 250 mg/ml had a bacteriostatic effect. The mean minimum inhibitory concentration (MIC) for studied *Lactobacillus* strains was determined as 1000 mg/ml.

*Conclusion:* High concentrations of clindamycin achieved in the vagina by intravaginal application might be inhibitory for *Lactobacillus* .


*Infect Dis Obstet Gynecol 2001;9:239–244*

### The inhibitory effect of clindamycin on *Lactobacillus in vitro*

**Alla Aroutcheva[1], Jose A. Simoes[1], Susan Shott[1], and Sebastian Faro[2]**

[1]*Department of Obstetrics and Gynecology, Rush-Presbyterian-St. Luke's Medical Center, Chicago, IL*
[2]*Department of Obstetrics, Gynecology and Reproductive Medicine, University of Texas Houston Health Science Center, and The Woman's Hospital of Texas, Houston, TX*

***Objective:*** To evaluate the *in vitro* effect of varying concentrations of clindamycin on *Lactobacillus* spp.
***Methods:*** Concentrations of clindamycin ranging from 1.95–20 000 μg/ml were studied for their effect on the growth of six strains of *Lactobacillus*.
***Results:*** Clindamycin concentrations between 1.95–31.25 μg/ml had no statistically significant effect on growth of lactobacilli ($p > 0.05$). Concentrations 125 and 250 μg/ml had a bacteriostatic effect. The mean minimum inhibitory concentration (MIC) for studied *Lactobacillus* strains was determined as 1000 μg/ml.
***Conclusion:*** High concentrations of clindamycin achieved in the vagina by intravaginal application might be inhibitory for *Lactobacillus*.

Key words: CLINDAMYCIN; *LACTOBACILLUS*; INHIBITION; *IN VITRO*

*Lactobacillus* appears to be a major factor in maintaining a balanced endogenous microflora. The production of hydrogen peroxide, bacteriocin and organic acids including lactic acid, act by suppressing the growth of the endogenous bacteria of the vaginal ecosystem.

A disturbance in the balance of the vaginal ecosystem results in an alteration of the endogenous vaginal microflora. One possible scenario is a decrease in the numbers of *Lactobacillus* spp. and an increase in the numbers of *Gardnerella vaginalis* and anaerobes. This shift to non-*Lactobacillus*-dominated vaginal flora results in an increase of *G. vaginalis* and anaerobes that leads to the development of bacterial vaginosis (BV).

BV is a significant alteration of the vaginal microflora because it is associated with a variety of pelvic infections in both the obstetric and gynecologic patient[1–3]. However, restoration of a healthy vaginal microflora in patients with BV has been difficult. The most common treatment regimens are oral or intravaginal metronidazole, or intravaginal clindamycin.

Clindamycin is frequently used in the treatment of BV in nonpregnant women[4–11]. The goal of effective BV treatment is not only inhibition of *G. vaginalis* and anaerobe growth, but also avoidance of a negative impact on the growth of *Lactobacillus* spp. One aim of BV treatment is to allow *Lactobacillus* to regain dominance and restore the vaginal ecosystem to a healthy state.

Previous clinical studies of intravaginal clindamycin showed initial suppression of lactobacilli growth. But growth and dominance of *Lactobacillus* was restored in a month[12,13].

The *in vitro* study conducted by Bayer and colleagues[14] determined that 98% of *Lactobacillus* strains were inhibited by clindamycin. The present

Correspondence to: Alla Aroutcheva, Department of Obstetrics and Gynecology, Rush-Presbyterian-St. Luke's Medical Center, Chicago, IL

study was performed to evaluate the *in vitro* effect of varying concentrations of clindamycin on *Lactobacillus* spp. High concentrations are achieved in the vagina when intravaginal clindamycin (2%) cream is used to treat BV.

### MATERIALS AND METHODS

### Bacterial isolates

*Lactobacillus* spp. were obtained from the collection maintained in the Infectious Diseases Laboratory in the Department of Obstetrics and Gynecology at Rush-Presbyterian-St. Luke's Medical Center. *Lactobacillus* spp. were originally isolated from the lower genital tract of patients with a normal vaginal ecosystem who attended the private practice of one of the investigators. Each patient gave her consent allowing a specimen to be obtained by swabbing the middle lateral vaginal wall. Lactobacilli were isolated by inoculating MRS agar (Remel, Lenexa, Kansas) incubated anaerobically, at 36°C for 24–48 h. *Lactobacillus* spp. were identified using the Biolog Identification System (Biolog Inc, Hayward, CA).

The following vaginal strains of lactobacilli were used in this study: one *L. acidophilus*, two *L. casei ss casei*, two *L. casei ss rhamnosus* and one *L. jensenii*.

### Antimicrobial agent and susceptibility test

Clindamycin powder suitable for susceptibility testing was obtained from the Sigma Chemical Co, St. Louis, MO. The minimum inhibitory concentration (MIC) was determined by the broth microdilution method. MRS broth was the medium used for susceptibility testing. Clindamycin dilutions were prepared to a concentration of 40 mg/ml in MRS broth and further diluted in the same medium. Serial dilutions were made in a range of 1.95–20 000 μg/ml.

Inoculum was prepared from overnight growth of *Lactobacillus* on MRS agar. Several colonies were picked with a cotton swab and suspended in sterile phosphate buffered saline (PBS) to achieve a turbidity of 0.5 McFarland standard. This suspension was further mixed with MRS broth at 1:100 dilution. We added 150 μl of inoculum to the microplate wells containing the same volume of clindamycin solutions in MRS broth. As a control, growth of each *Lactobacillus* strain in MRS broth was used. An anaerobic environment in wells was created with paraffin oil. Growth of lactobacilli in different concentrations of clindamycin was monitored using a reader-incubator Bioscreen C Analyser System (Labsystem, Helsinki, Finland). Lactobacilli were grown at 37°C with periodic shaking. The optical density (OD), as a reflection of growth, was measured automatically every four hours for 48 h.

### Statistical analysis

The difference between growth of lactobacilli and controls in clindamycin concentrations was calculated using the Freidman test. The standard deviation was calculated for range of growth.

### RESULTS

The susceptibility data for clindamycin against six tested strains of *Lactobacillus* collected from the Bioscreen reader was statistically analyzed (Table 1). No significant difference in the growth rate versus control was found in the concentration range 1.95–31.25 μg/ml ($p > 0.05$) (Figure 1).

Figure 2 shows different inhibition effects in 24 and 48 h. No pronounced inhibition was observed with concentrations at or below 31.5 μg/ml. Growth was inhibited up to 12 h incubation at a concentration of 62.5 μg/ml, but after 24 h growth resumed and was comparable to that of the control. Inhibition at 12 h was 88.5% and at 24 and 48 h was 36% and 18.2% respectively. Almost 100% of growth was inhibited at 125 μg/ml concentration within 24 h. Over the next 24 h the bacteriostatic effect abated and growth resumed. Clindamycin at a concentration of 250 μg/ml had a similar effect to 125 μg/ml on growth of lactobacilli. Inhibition of growth was observed for 28 h at a concentration 500 μg/ml.

Complete growth inhibition occurred at concentrations of 1000 μg/ml and above (Figure 3). The effective MIC varied for different species. The MIC for two strains of *L. casei ss rhamnosus*, one strain of *L. casei ss casei* and one strain of *L. jensenii*, was 1000 μg/ml. *L. acidophilus* was inhibited with

**Table 1** Growth of *Lactobacillus* (OD) (mean ± SD) with different clindamycin concentrations.

| | *Clindamycin concentration* | | | | | | | |
|---|---|---|---|---|---|---|---|---|
| *Time (h)* | *0* | *31.25 μg/ml* | *62.5 μg/ml* | *125 μg/ml* | *250 μg/ml* | *500 μg/ml* | *1000 μg/ml* | *3000 μg/ml* |
| 12* | 1.68 ± 0.4 | 1.27 ± 0.33 | 0.91 ± 0.28 | 0.69 ± 0.01 | 0.68 ± 0.02 | 0.67 ± 0.01 | 0.67 ± 0.01 | 0.67 ± 0.01 |
| | 1.83 | 1.34 | 0.83 | 0.68 | 0.68 | 0.68 | 0.67 | 0.67 |
| 24 | 1.82 ± 0.53 | 1.68 ± 0.49 | 1.36 ± 0.52 | 0.89 ± 0.33 | 1.01 ± 0.29 | 0.67 ± 0.01 | 0.66 ± 0.01 | 0.66 ± 0.01 |
| | 1.95 | 1.87 | 1.52 | 0.68 | 1.08 | 0.67 | 0.66 | 0.67 |
| 36 | 1.72 ± 0.49 | 1.62 ± 0.47 | 1.50 ± 0.42 | 1.21 ± 0.44 | 1.33 ± 0.52 | 0.79 ± 0.21 | 0.66 ± 0.01 | 0.66 ± 0.01 |
| | 1.80 | 1.72 | 1.68 | 1.35 | 1.59 | 0.70 | 0.66 | 0.66 |
| 48 | 1.70 ± 0.46 | 1.59 ± 0.46 | 1.50 ± 0.44 | 1.30 ± 0.35 | 1.34 ± 0.51 | 1.18 ± 0.47 | 0.66 ± 0.01 | 0.66 ± 0.01 |
| | 1.79 | 1.67 | 1.62 | 1.47 | 1.62 | 1.26 | 0.66 | 0.66 |

*Mean value of initial OD for 0 time was 0.7

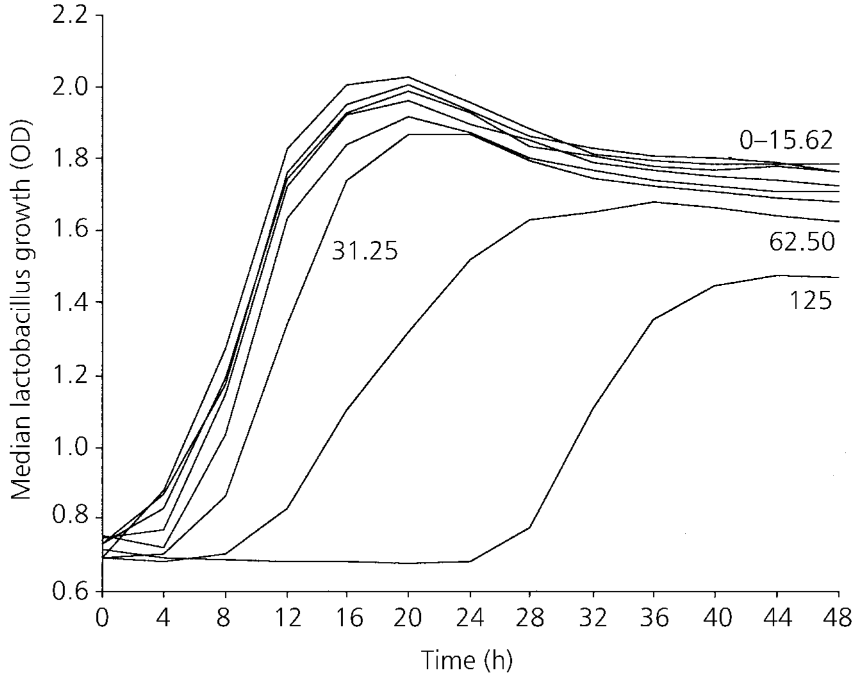

**Figure 1** Median *Lactobacillus* growth in clindamycin of varying concentrations. Concentrations are in μg/ml. Control = 0 μg/ml

500 μg/ml, and the MIC for one strain of *L. casei ss casei* was 250 μg/ml.

### DISCUSSION

Clindamycin is reported to be one of the most effective antimicrobial agents for the treatment of BV. Clindamycin is active against anaerobic microflora, and is effective against approximately 95% of the species, *Bacteroides*, *Fusobacterium* and 70–84% of *Peptostreptococcus* sp. The $MIC_{90}$ range for anaerobes is 0.007–1.6 μg/ml[15]. Clindamycin was found to be highly active against *G. vaginalis* with $MIC_{90}$ 0.016–0.19[16], 0.6 μg/ml[17] and 0.06–2 μg/ml[18].

Although clindamycin is believed to have high efficacy in the treatment of BV, published data shows that neither clindamycin nor metronidazole is able to eradicate BV-associated bacteria[13]. After treatment for BV, many women remained colonized by *G. Vaginalis* or *Bacteroides* sp. even though Gram's stain and clinical criteria no longer indicated the presence of BV[19].

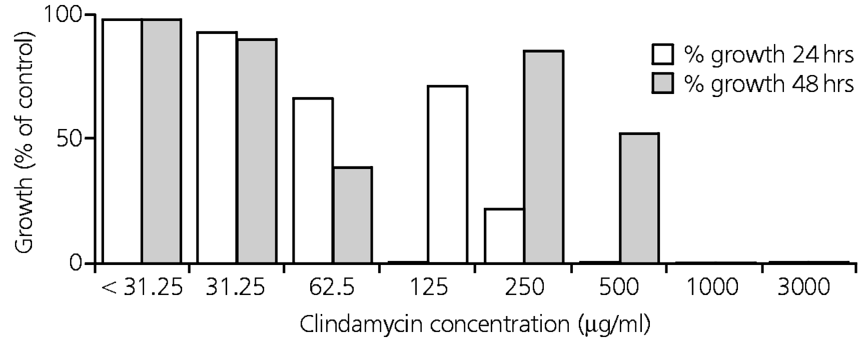

**Figure 2** Growth of *Lactobacillus* spp. at 24 h and 48 h with different clindamycin concentrations. Growth is shown as a percentage of the control (0 μg/ml clindamycin)

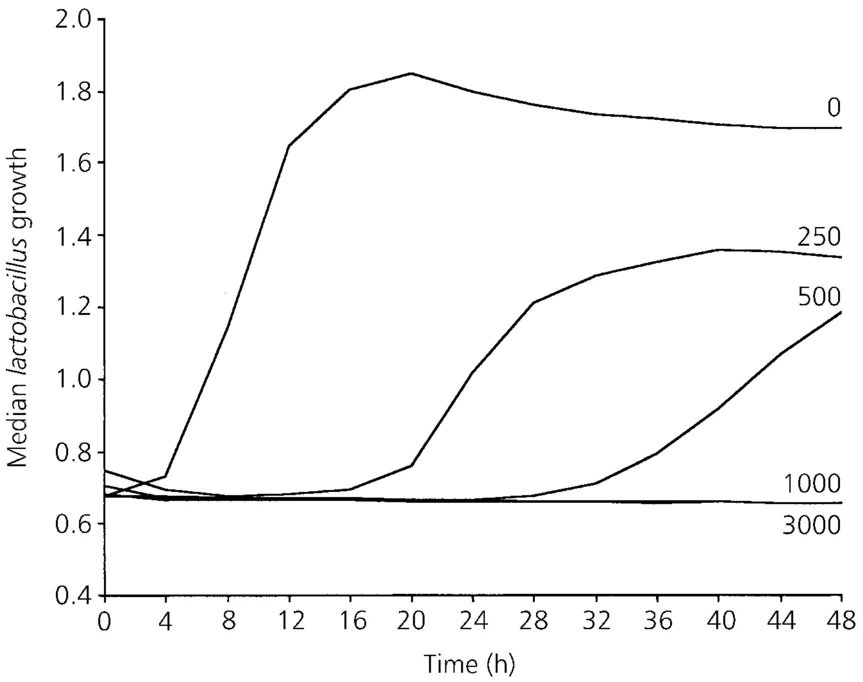

**Figure 3** Median *Lactobacillus* growth with various clindamycin concentrations. Values are in μg/ml. The control is 0 μg/ml

Ferris and colleagues[20] demonstrated that clindamycin vaginal cream, by culture criteria, effectively treated BV in 86.2% of the cases. They performed DNA tests and found that *G. vaginalis* still remained after treatment. In another study, intravaginal treatment of BV with clindamycin cream did not reduce preterm delivery or low birth weight infants[21]. Even though the microbiological characteristic of BV is overgrowth by *G. vaginalis* and anaerobes, colonization by *Lactobacillus* spp. still occurs. Spiegel and co-workers[22] used gas-liquid chromatography to recover lactobacilli in 23.5% of women with nonspecific vaginitis.

We observed suppressed growth of *Lactobacillus* spp. in 46.1% of BV cases. Furthermore, half of the *Lactobacillus* spp. did not grow on MRS agar (the selective medium for lactic acid bacteria), but grew on HBT agar (the bilayer agar with human blood and Tween 80), which is a selective medium for *G. vaginalis*.

There is always concern that treatment of BV will suppress the growth of lactobacilli and prevent reconstitution of the normal vaginal microflora. Clindamycin is usually applied intravaginally, as a 2% vaginal cream for 7 days or with vaginal ovules for 3 days. The single dose of both forms contains 100 mg clindamycin. Cleocin vaginal ovules, 3 days therapy, were found to be as effective for BV treatment as 7 days oral metronidazole (68.1% versus 66.7%) and 7 days Cleocin vaginal cream (66.0% versus 59.6%) (data on file, Pharmacia & Upjohn Company). Absorption of clindamycin from a suppository is equivalent to about 30 mg per day (Data on file, Pharmacia & Upjohn Company).

During topical application of clindamycin, vaginal lactobacilli are exposed to a high concentration of antibiotic. Our *in vitro* study shows that growth of lactobacilli was inhibited with concentrations of clindamycin ranging between 250–1000 μg/ml, with a mean MIC of 1000 μg/ml, which is 100 times lower than a dose of intravaginal clindamycin. Lactobacilli, suppressed by an abundant growth of abnormal microflora, might be further suppressed by a high concentration of clindamycin. Suppression of normal bacterial flora and overgrowth of *E. coli* and *Enterococci* was a result of clindamycin vaginal cream therapy[11]. Replacement of bacterial dominance by bacteria, such as *E. coli*, may place the patient at significant risk of infection.

Total inhibition of lactobacilli growth can result with doses of clindamycin that are lower than that topically administered, as shown in this study. The partial inhibition of other endogenous vaginal bacteria growth by intravaginal administration of clindamycin may offer insight into why there is a significant recurrence of BV in patients receiving treatment.

### REFERENCES

1. Gibbs RS. Chorioamnionitis and bacterial vaginosis. *Am J Obstet Gynecol* 1993;169:460–2
2. Krohn MA, Hillier SL, Nugent RP, *et al.* The genital flora of women with intraamniotic infection. Vaginal Infection and Prematurity Study Group. *J Infect Dis* 1995;171:1475–80
3. Larsson PG, Platz-Christensen JJ, Thejls H, *et al.* Incidence of pelvic inflammatory disease after first-trimester legal abortion in women with bacterial vaginosis after treatment with metronidazole: a double-blind, randomized study. *Am J Obstet Gynecol* 1992;166:100–3
4. Borin MT, Powley GW, Tackwell KR, *et al.* Absorption of clindamycin after intravaginal application of clindamycin phosphate 2% cream. *J Antimicrob Chemother* 1995;35:833–41
5. Dhar J, Arya OP, Timmins DJ, *et al.* Treatment of bacterial vaginosis with a three day course of 2% clindamycin vaginal cream: a pilot study. *Genitour Med* 1994;70:121–3
6. Fischbach F, Petersen EE, Weissenbacher ER, *et al.* Efficacy of clindamycin vaginal cream versus oral metronidazole in the treatment of bacterial vaginosis. *Obstet Gynecol* 1993;82:405–10
7. Hill GB, Livengood CH. Bacterial vaginosis-associated microflora and effects of topical intravaginal clindamycin. *Am J Obstet Gynecol* 1994; 171:1198–204
8. Larsson PG, Platz-Christensen JJ, Dalaker K, *et al.* Treatment with 2% clindamycin vaginal cream prior to first trimester surgical abortion to reduce signs of postoperative infection: a prospective, double-blinded, placebo-controlled, multicenter study. *Act Obstet Gynecol Scand* 2000;79:390–6
9. Paavonen J, Mangioni C, Martin MA, *et al.* Vaginal clindamycin and oral metronidazole for bacterial vaginosis: a randomized trial. *Obstet Gynecol* 2000; 96:256–60
10. Schlicht JR. Treatment of bacterial vaginosis. *Ann Pharmaco* 1994;28:483–7
11. Sweet RL. New approaches for the treatment of bacterial vaginosis. *Am J Obst Gynecol* 1993;169: 479–82
12. Agnew KJ, Hillier SL. The effect of treatment regimens for vaginitis and cervicitis on vaginal colonization by lactobacilli. *Sex Trans Dis* 1995; 22:269–73
13. Flores-Rivera E, Casanova-Roman G, Beltran M, *et al.* Bacterial vaginosis. Relation between the vaginal flora and the vaginal epithelial cells under different treatments. Ultrastructural study. *Ginecol Obstet Mex* 1997;65:182–90
14. Bayer AS, Chow AW, Concepcion N, *et al.* Susceptibility of 40 lactobacilli to six antimicrobial agents with broad gram-positive anaerobic spectra. *Antimicrob Agen Chemother* 1978;14:720–2
15. Summanen P, Barron EJ, Citron DM, *et al.* *Wadsworth anaerobic bacteriology manual* 5th edn. 1993 Star, Los Angeles, CA

16. Altrichter T, Heizmann WR. *Gardnerella vaginalis*: transport, microscopy, testing resistance. *Geburtsh Frauen* 1994;54:606–11
17. Shanker S, Toohey M, Munro R. *In vitro* activity of seventeen antimicrobial agents against *Gardnerella vaginalis*. *Euro J Clin Microbiol* 1982;1:298–300
18. Weber P, Dubois S, and Boussougant Y. *In vitro* activity of pristinamycin and its components against gram-negative anaerobic bacilli and *Gardnerella vaginalis*. *J Anti Chemo* 1989;23:825–3019
19. Hillier S, Krohn MA, Watts DH, *et al.* Microbiologic efficacy of intravaginal clindamycin cream for the treatment of bacterial vaginosis. *Obstet Gynecol* 1990;76:407–13
20. Ferris DG, Litaker MS, Woodward L, *et al.* Treatment of bacterial vaginosis: a comparison of oral metronidazole, metronidazole vaginal gel, and clindamycin vaginal cream. *J Fam Pract* 1995;41: 443–9
21. Joesoef MR, Hillier SL, Wiknjosastro G, *et al.* Intravaginal clindamycin treatment for bacterial vaginosis: effects on preterm delivery and low birth weight. *Amer J Obstet Gynecol* 1995;173:1527–31
22. Spiegel CA, Amsel R, Eschenbach D, *et al.* Anaerobic bacteria in nonspecific vaginitis. *N Engl J Med* 1980;303:601–7

RECEIVED 06/18/01; ACCEPTED 10/2/01
